# Pyrene-Based Blue AIEgen: Enhanced Hole Mobility and Good EL Performance in Solution-Processed OLEDs

**DOI:** 10.3390/molecules22122144

**Published:** 2017-12-04

**Authors:** Jie Yang, Jianwen Qin, Zichun Ren, Qian Peng, Guohua Xie, Zhen Li

**Affiliations:** 1 Hubei Key Lab on Organic and Polymeric Opto-Electronic Materials, Department of Chemistry, Wuhan University, Wuhan 430072, China; jieyangwhu@whu.edu.cn (J.Y.); qinjianwen@whu.edu.cn (J.Q.); renzichun225@163.com (Z.R.); 2 Key Laboratory of Organic Solids, Beijing National Laboratory for Molecular Science (BNLMS), Institute of Chemistry, Chinese Academy of Sciences, Beijing 100190, China; qpeng@iccas.ac.cn

**Keywords:** aggregation-induced emission, blue emitter, hole-transporting ability, solution-processed OLED

## Abstract

Organic luminogens with strong solid-state emission have attracted much attention for their widely practical applications. However, the traditional organic luminogens with planar conformations often suffer from the notorious aggregation-caused quenching (ACQ) effect in solid state for the π–π stacking. Here, a highly efficient blue emitter TPE-4Py with an aggregation-induced emission (AIE) effect is achieved by combining twisted tetraphenylethene (TPE) core and planar pyrene peripheries. When the emitter was spin-coated in non-doped OLEDs with or without a hole-transporting layer, comparable EL performance was achieved, showing the bifunctional property as both an emitter and a hole-transporting layer. Furthermore, its EL efficiency was promoted in doped OLED, even at a high doping concentration (50%), because of its novel AIE effect, with a current efficiency up to 4.9 cd/A at 484 nm.

## 1. Introduction

Organic light-emitting diodes (OLEDs) have drawn increasing interest for their wide applications in full-color display and white light illumination [1,2]. As one kind of important emitting materials, efficient blue-emitting luminogens are urgently needed for the perfect commercialization of OLEDs [3,4]. However, traditional luminogens with planar conformations often suffer from an aggregation-caused quenching (ACQ) effect for the intermolecular π–π stacking, thus resulting in inferior EL performance. To conquer the ACQ effect and achieve outstanding EL performance, the development of aggregation-induced emission (AIE) luminogens (a series of propeller-like luminogens were found to be non-luminescent in the solution but highly emissive in the aggregation state, first termed by Benzong Tang et al.) should be explored [5,6].

Since first synthesized by Weitzenböck in 1913, pyrene and its derivatives have attracted particular attention in the research of blue emissive materials for their high quantum efficiency and good hole-transporting ability [7,8]. However, their tendency to form excimer or exciplex in a solid state, resulting from their plate-like structures, often leads to the large red-shifted emission or aggregation-caused ACQ effect [9,10]. In order to conquer the ACQ effect and realize the efficient blue emission of pyrene derivatives, great attempts have been made in our previous works. Mainly, two strategies have been developed. One is to decorate the pyrene core with the periphery groups in twisted conformation (tetraphenylethene, *m*-terphenyl, 9,9-diphenyl fluorene et al.) (Chart S1) [11,12]. With it, highly efficient blue AIEgens can be obtained, as can good EL performances with a current efficiency and external quantum efficiency up to 2.94 cd A^−1^ and 3.46% at CIE coordinates of (0.15, 0.09). However, the periphery groups in a twisted conformation may severely hinder the interaction of pyrene cores, thus resulting in inferior hole-transporting ability. Later, another strategy was utilized, with an AIE building block acting as a core and pyrene as a peripheral group, where a series of efficient blue materials was developed with enhanced hole-transporting ability, achieving a good interaction between pyrene peripheries (Chart S2) [13]. When emitters are fabricated in OLEDs with or without a hole-transporting layer, they exhibit comparable EL performances, with a current efficiency and external quantum efficiency up to 4.64 cd A^−1^ and 2.79% at CIE coordinates of (0.16, 0.21), respectively, showing bifunctional properties. Regardless of their good performance, all of the attempts have been centered on the OLED via vapor deposition under vacuum. However, the thermal evaporation process suffers from fabrication complexity and low utilization of expensive light-emitting materials. On the other hand, a solution process has been considered to be a better way of improving process efficiency and reducing production cost [14]. For solution-processed OLEDs, an emitting layer is generally spin-coated on a wet-processed PEDOT:PSS (poly(3,4-ethylenedioxythiophene):poly(styrenesulfonate)) layer without any hole-transporting material, because spin coating the emitting layer may dissolve the underlying hole-transporting layer. Thus, it would be much better to develop a blue material with both a high PL efficiency and a good hole-transporting ability [15].

In this communication, a highly efficient blue emitter TPE-4Py with an AIE effect is achieved by combining twisted tetraphenylethene (TPE) core and four planar pyrene peripheries. It was found to show an enhanced hole-transporting ability in light of the good interaction between the pyrene side groups. When the emitter was spin-coated in non-doped OLEDs with or without a hole-transporting layer, comparable EL performance was achieved. Furthermore, its EL efficiency was promoted in doped OLED, even at a high doping concentration (50%), because of its novel AIE effect, with a current efficiency up to 4.9 cd/A at 484 nm. Herein, we present in detail the synthesis, characterizations, theoretical calculations, and light emitting properties of TPE-4Py.

## 2. Results and Discussion

TPE-4Py was synthesized via Suzuki coupling reaction with a moderate yield of 30% (Scheme S1). The structure of TPE-4Py was characterized via ^1^H, ^13^C NMR, mass spectroscopy, and elemental analysis (Figure S1). Its thermal properties were then investigated by thermal gravimetric analysis (TGA) and differential scanning calorimetry (DSC). As shown in Figure S2, it was thermally stable with the thermal decomposition temperature (*T*_d_, corresponding to 5% weight loss) and glass transition temperature (*T*_g_) up to 494 °C and 109 °C respectively, for the introduction of rigid pyrene groups.

**Figure 1:**
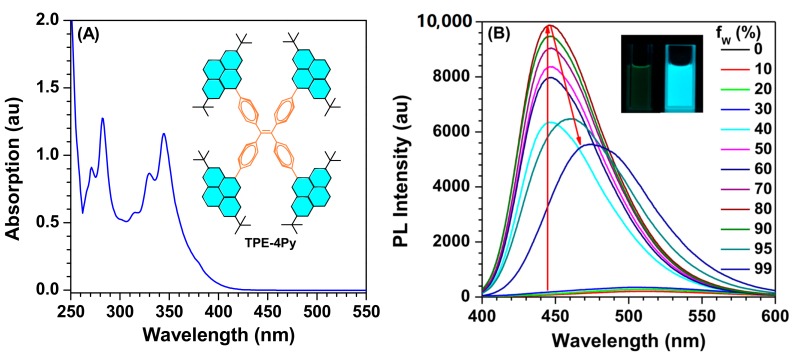
(**A**) UV-vis absorption spectrum of TPE-4Py in THF solution. Concentration: 10 µM, inset: molecular structure of TPE-4Py; (**B**) The PL spectra of TPE-4Py in THF/H_2_O mixtures with different water fractions. Concentration: 10 µM; excitation wavelength: 330 nm; inset: photographs of theTPE-4Py in the THF/H_2_O mixtures (*f*_W_ = 0% and 90%) taken under the illumination of a 365 nm UV lamp.

Because of the introduced tert-butyl groups, TPE-4Py has good solubility in common organic solvents, such as chloroform and tetrahydrofuran (THF) et al., but is insoluble in water. Figure 1A shows its UV-vis absorption spectrum with a maximum absorption peak at 344 nm (Figure 1A). From the onset of the absorption spectrum, its energy band gap was calculated to be as high as 3.2 eV, indicating limited conjugation. With the aid of cyclic voltammetry, its HOMO energy is derived from the onset potential of oxidation of 5.5 eV, subtracting the optical band gap energy from a LUMO energy of 2.3 eV (Figure S3).

**Figure 2:**
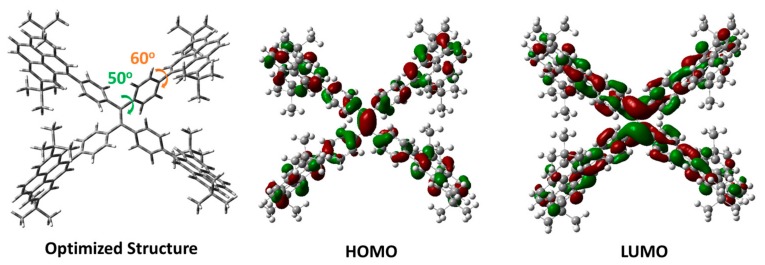
Optimized molecular structure and calculated molecular orbital amplitude plots of HOMO and LUMO levels of TPE-4Py.

To study the effect of aggregation on its light emission process, the photoluminescence (PL) spectra in THF/water mixtures were measured (Figure 2B). In dilute THF solution, negligible emission was observed with the PL curve nearly parallel to the abscissa, due to the intramolecular motion. When a moderate amount of water (40 < *f*_W_ < 80) was added to the THF solution, it could give a much more enhanced PL intensity with the deep blue emission peaked at 446 nm for the formation of aggregation, indicating a typical AIE effect [16,17]. However, slightly decreased and red-shifted emissions would occur after a large amount of water (*f*_W_ > 90) was added, which should originate from the transition from a crystal to amorphous state [18]. Furthermore, the absolute quantum yield of TPE-4Py powder was measured to be as high as 50% because of its novel AIE effect. On the other hand, its fluorescent lifetime was just 2.8 ns and this short lifetime could certify the absence of excimer or exciplex in the solid state (Figure S4) [19].

To investigate the structure–property relationship of TPE-4Py, Density Functional Theory (DFT) calculations (B3LYP/6-31g*) were carried out. As shown in Figure 2, the optimized structure shows a heavily twisted conformation with a torsion angle of about 50° for TPE, while the dihedral angle between pyrene and the adjacent phenyl ring was as wide as 60°. Thus, the twisted conformation could lead to weak conjugation as well as blue emission. Furthermore, it could restrict the π–π stacking effectively, resulting in an abnormal AIE effect. The electron clouds in both HOMO and LUMO levels distribute on the whole molecule, and good overlap of the frontier orbitals was achieved, which would be beneficial for the high PL efficiency in a solid state.

**Figure 3:**
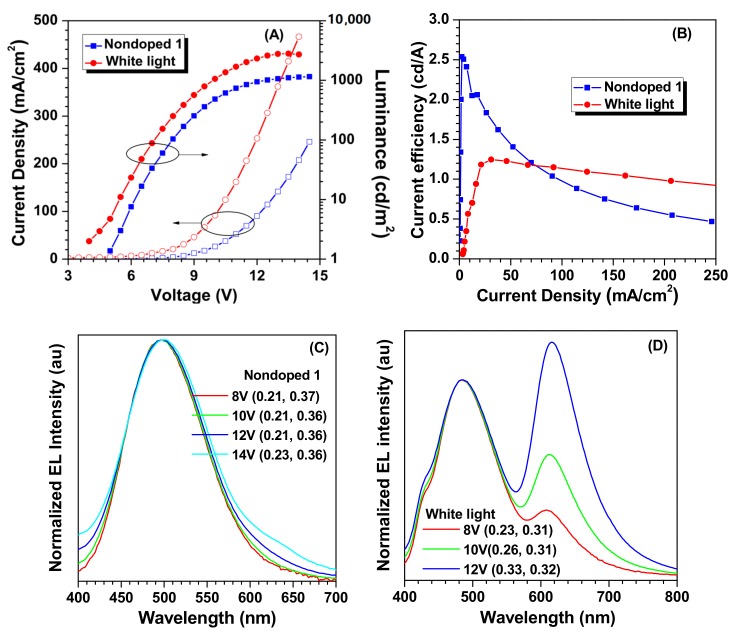
Changes in (**A**) current density and luminance with the applied voltage; (**B**) current efficiency with the current density; (**C**) EL spectra with the voltage in Nondoped 1; (**D**) EL spectra with the voltage in white light device. Device configuration: Nondoped 1: ITO/PEDOT:PSS (30 nm)/Poly-TPD (30 nm)/TPE-4Py (30 nm)/TPBi (50 nm)/Liq (1 nm)/Al (100 nm); white light: ITO/PEDOT:PSS (30 nm)/Poly-TPD (30 nm)/mCP:OXD-7:TPE-4Py (70:20:10, 30 nm)/TPBi (50 nm)/Liq (1 nm)/Al (100 nm).

Good solubility and thermal stabilities, as well as efficient light emissions in the solid state of TPE-4Py, prompted us to investigate its electroluminescence properties with solution-processed OLED. Firstly, a non-doped OLED device (Nondoped 1) with a configuration of ITO/PEDOT:PSS (30 nm)/Poly-TPD (30 nm)/TPE-4Py (30 nm)/TPBi (50 nm)/Liq (1 nm)/Al (100 nm) was fabricated, in which PEDOT:PSS, Poly-TPD, and TPBi worked as the hole-injection, hole-transporting, and electron-transporting layers, respectively, and TPE-4Py served as the emitting layer. Figure 3 and Figure S5 show the current density–voltage–brightness (*J–V–L*) characteristics, EL spectra, current efficiency, external quantum efficiency, and power efficiency versus the current density curves. As listed in Table 1, moderate EL performance was achieved for Nondoped 1 with a current efficiency and external quantum efficiency up to 2.54 cd A^−1^ and 1.08% at CIE coordinates (0.21, 0.36), respectively. In comparison with the PL emission (468 nm) in thin film, red-shifted emission was observed in its EL spectrum (498 nm), indicating that it mainly existed in an amorphous state in the OLED device (Figure S6). On the other hand, an exciplex host (mCP:OXD-7) was added to construct a doped OLED with a white emission. As shown in Figure 3D, its CIE coordinates were (0.33, 0.32) at 12 V, much closer to the value of the theoretical white point (0.33, 0.33), while the current efficiency was 1.25 cd A^−1^.

**Figure 4:**
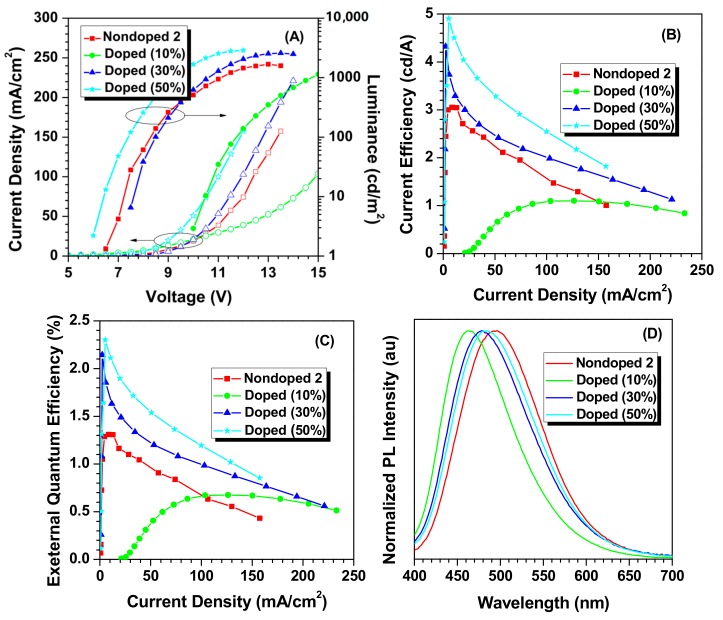
Changes in (**A**) current density and luminance with the applied voltage; (**B**) current efficiency with the current density; (**C**) external quantum efficiency with the current density; and (**D**) EL spectra with voltage. Device configuration: Nondoped 2: ITO/PEDOT:PSS (30 nm)/TPE-4Py (30 nm)/TmPyPB (50 nm)/Liq (1 nm)/Al (100 nm); Doped: ITO/PEDOT:PSS (30 nm)/mCP:TPE-4Py (10% or 30% or 50%, 30 nm)/TmPyPB (50 nm)/Liq (1 nm)/Al (100 nm).

To further explore the hole-transporting characteristic of TPE-4Py, another nondoped device (Nondoped 2) was constructed with the configuration of ITO/PEDOT:PSS (30 nm)/TPE-4Py (30 nm)/TmPyPB (50 nm)/Liq (1 nm)/Al (100 nm), in which the hole-transporting layer Poly-TPD was eliminated, and the electron transporting layer was replaced by TmPyPB, which has greater electron mobility (1 × 10^−3^ cm^2^ V^−1^ s^−1^) [20,21]. Excitingly, better EL performance was obtained with a current efficiency and external quantum efficiency up to 3.05 cd A^−1^ and 1.31% at CIE coordinates (0.21, 0.36), respectively, for the more balanced carrier transport and exciton recombination (Figure 4, Figure S7). The present results demonstrate that combining pyrene peripheries and an AIE core would be an effective way to develop good hole-transporting materials with the AIE property, achieving a good interaction between pyrene side groups.

Later, doped OLEDs based on TPE-4Py were constructed with device configurations of ITO/PEDOT:PSS (30 nm)/mCP:TPE-4Py (10% or 30% or 50%, 30 nm)/TmPyPB (50 nm)/Liq (1 nm)/Al (100 nm), in which mCP served as host material and TPE-4Py as guest one. Because of the abnormal AIE effect of TPE-4Py, the EL efficiency promotion, rather than roll-off, was achieved by increasing the doping concentration from 10 to as high as 50%. Among the doped ones fabricated, the device employing a 50% doping concentration as the emitting layer could show a sky blue emission (484 nm) with a maximum current efficiency and external quantum efficiency up to 4.9 cd A^−1^ and 2.3%, respectively, which is among the best EL performances for solution-processed OLEDs based on blue AIEgens ever observed [22,23,24,25].

## 3. Conclusions

In summary, a highly efficient blue emitter of TPE-4Py with an aggregation-induced emission (AIE) effect was constructed by combining an AIE core and planar peripheries. Because of the introduction of pyrene peripheral groups, the target compound shows an enhanced hole-transporting ability. When the emitter was spin-coated in non-doped OLEDs with or without a hole-transporting layer, a comparable EL performance was achieved with a current efficiency up to 3.05 cd A^−1^ at 496 nm. On the other hand, pure white emission (0.33, 0.32) was achieved in a doped OLED with an exciplex as host. Furthermore, its EL efficiency was promoted in the doped OLED, even at a high doping concentration, because of its novel AIE effect. The doped device employing a 50% doping concentration as the emitting layer gave a current efficiency up to 4.9 cd/A at 484 nm, which is among the best EL performance for solution-processed OLEDs based on blue AIEgens. It is anticipated that more and more efficient blue luminogens with enhanced carrier mobility will be developed through this strategy.

## Figures and Tables

**Table 1:** The EL performance of TPE-4Py.

TPE-4Py	*λ*_EL_	*V*_on_	*L*_max_	*η*_P, max_	*η*_C, max_	*η*_ext, max_	CIE
	(nm)	(V)	(cd m^−2^)	(lm W^−1^)	(cd A^−1^)	(%)	(x, y)
**Nondoped 1**	498	5.0	1153	1.06	2.54	1.08	0.21, 0.36
**White light**	/	4.0	2800	0.46	1.25	0.70	0.33, 0.32
**Nondoped 2**	496	6.5	1678	1.00	3.05	1.31	0.21, 0.36
**Doped 10**	462	10.0	1955	0.23	1.10	0.67	0.18, 0.22
**Doped 30**	480	7.5	2581	1.60	4.32	2.14	0.20, 0.29
**Doped 50**	484	6.0	2862	1.93	4.90	2.30	0.20, 0.32

Abbreviations: *V*_on_ = turn-on voltage at 1 cd m^−2^; *L*_max_ = maximum luminance; *η*_P, max_, *η*_C, max_, and *η*_ext, max_ = maximum power, current, and external efficiencies, respectively. CIE = Commission International de l’Eclairage coordinates.

## Supplementary Materials

Supplementary Materials are available online: synthesis, TGA, DSC, cyclic voltammetry curve, PL decay curve, and EL performance.
