# Supplementary material for: Pyrene-Based Blue AIEgen: Enhanced Hole Mobility and Good EL Performance in Solution-Processed OLEDs
Source: Molecules. 2017 Dec 4;22(12):2144. doi: 10.3390/molecules22122144 (PMC6149785; doi:10.3390/molecules22122144)
Supplement: Supplementary file 1 [file molecules-22-02144-s001.pdf]

Supplemental Material

Pyrene based blue AIEgen: enhanced hole mobility and good EL performance in solution-processed OLED

Jie Yang[1], Jianwen Qin[1], Zichun Ren[1], Qian Peng[2], Guohua Xie[1,*] and Zhen Li[1,*]

Characterizations

^1H and ^{13}C NMR spectra were recorded on a Mercury vx300 spectrometer. Mass spectrum was measured on a MALDI-TOF mass spectrometer. Elemental analyses of carbon and hydrogen were performed on a Carlo-Erba-1106 microanalyzer. UV/Vis absorption spectrum was recorded on a Shimadzu UV-2500 recording spectrophotometer. Photoluminescence spectra were recorded on a Hitachi F-4600 fluorescence spectrophotometer. Differential scanning calorimetry (DSC) was performed on a NETZSCH DSC 200 PC instrument from room temperature to 250 °C at a heating rate of 10 °C min^{-1} under argon. Thermogravimetric analysis (TGA) was performed on a NETZSCH STA 449C instrument. The thermal stability of the sample under a nitrogen atmosphere was determined by measuring its weight loss while heating from 25 to 750 °C at a rate of 10 °C min^{-1}. Cyclic voltammetry (CV) was carried out on a CHI voltammetric analyzer in a three-electrode cell with a platinum counter electrode, an Ag/AgCl reference electrode, and a glassy carbon working electrode at a scan rate of 100 mV s^{-1} in anhydrous dichloromethane solution with 0.1 M tetrabutylammonium perchlorate (purchased from Alfa Aesar) as the supporting electrolyte and purged with nitrogen. The potential obtained in reference to the Ag/Ag^+ electrode was converted into value versus the saturated calomel electrode (SCE) by means of an internal ferrocenium/ferrocene (Fc^+/Fc) standard. The fluorescence quantum yield of powder was determined using a Hamamatsu C11347 Quantaurus-QY absolute fluorescence quantum yield spectrometer. Fluorescence lifetime was determined with a Hamamatsu C11367-11 Quantaurus-Tau time-resolved spectrometer.

Computational details

The geometrical and electronic properties were optimized at the B3LYP/6-31g* level using the Gaussian 09 program. The molecular orbitals were obtained at the same level of theory.

OLED device fabrication and measurement

The ITO surface was pre-cleaned in an ultrasonic solvent bath, and dried in an oven at 120 °C for more than 3 h, then treated with UV ozone for 15 min. After that, the substrates were transferred to the spin-coating process, and then different functional layers (PEDOT: PSS layer and hole-transporting layer and emissive layer) were sequentially spin-coated onto the substrates and were dried in vacuum. Then the samples were transferred to the deposition system, and electron-transporting layer and Liq/Al layers were sequentially deposited onto the substrate by thermal deposition in the vacuum of 10^{-6} Torr. The electroluminescent (EL) spectra were measured by a PR650 spectrometer. The current density-voltage-brightness (*J*-*V*-*R*) features of the OLEDs were measured with a Keithey 2400 Source meter and Konica Minolta

chromameter CS-200 with a calibrated silicon photodiode. The EQEs values were calculated from the current densities, current efficiencies, and the corresponding EL spectra. All measurements were carried out at room temperature under ambient conditions.

Synthesis

TPE-4Br (130 mg, 0.2 mmol), Py-4BO (440 mg, 1.0 mmol), $Pd(PPh_3)_4$ (0.10 g, 0.08 mmol) and potassium carbonate (553 mg, 4.0 mmol) in toluene (20 mL) and distilled water (7 mL) were added into a 200 mL Schlenk tube. The resultant mixture was refluxed for 3 days under argon, then extracted with dichloromethane. The combined organic extracts were dried over anhydrous Na_2SO_4 and concentrated by rotary evaporation. The crude product was purified by column chromatography on silica gel using chloroform/petroleum ether (v/v=1/5) as eluent. A light yellow solid was finally produced in the yield of 30% (95 mg). ^1H NMR (300 MHz, $CDCl_3$, δ): 8.27 (s, 4H), 8.19 (s, 8H), 8.13 (s, 4H), 8.06 (s, 4H), 8.04 (s, 8H), 7.62 (t, 16H), 1.28 (s, 72H). ^{13}C NMR (75Hz, $CDCl_3$, δ): 148.7, 148.2, 143.1, 140.6, 139.5, 139.3, 131.7, 130.9, 130.7, 130.4, 129.9, 129.5, 127.7, 127.6, 127.1, 123.1, 122.3, 122.0, 121.8, 121.0, 35.1, 31.9, 31.6, 29.6. MS (MALDI TOF), m/z: 1580.9053 ([M+], calcd for $C_{112}H_{116}$, 1580.9077). Anal. Calcd for $C_{112}H_{116}$: C, 92.61; H, 7.39. Found: C, 92.23; H, 7.47.

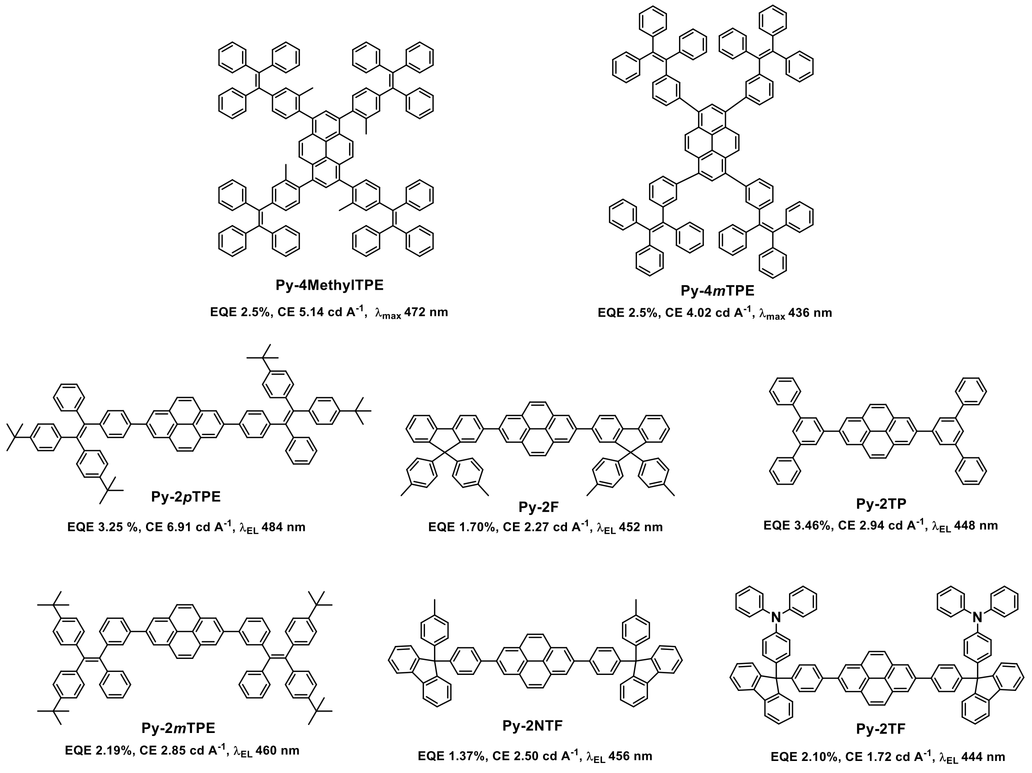

Chart S1. Blue AIEgens with pyrene core and twisted periphery groups in our previous report.

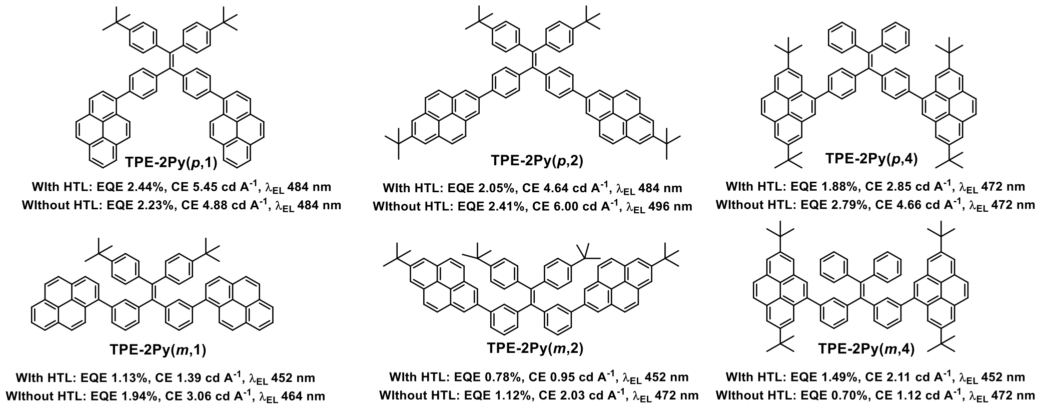

Chart S2. Blue AIEgens with TPE core and pyrene peripheries in our previous report.

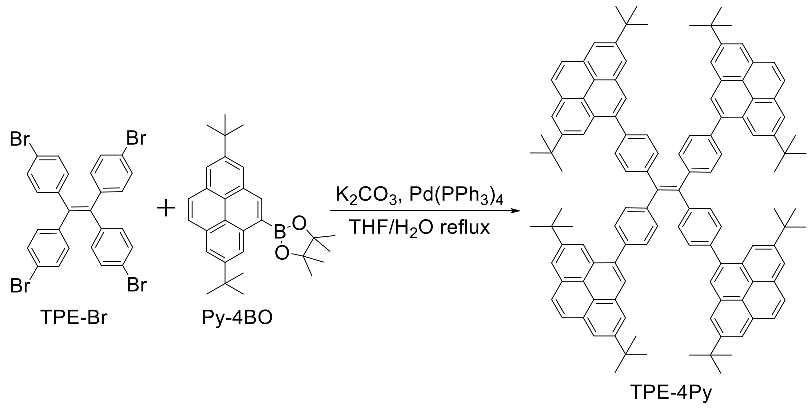

Scheme S1. Synthetic route for TPE-4Py.

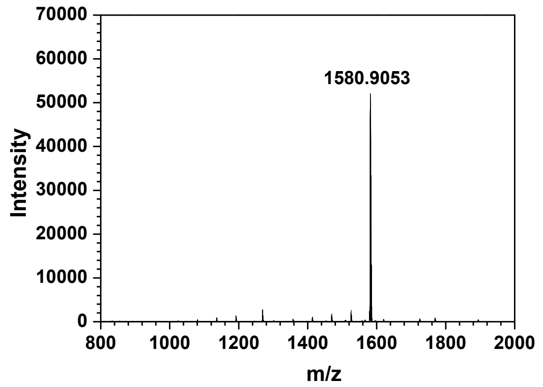

Figure S1. MALDI-TOF mass spectrum of TPE-4Py.

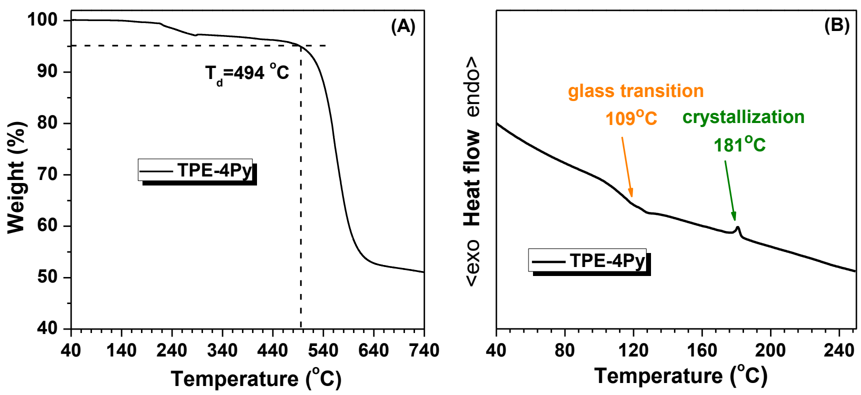

Figure S2. (A) TGA thermograms of TPE-4Py recorded at a heating rate of 10 °C/min; (B) DSC thermograms of TPE-4Py recorded under N_2 at a heating rate of 10 °C/min.

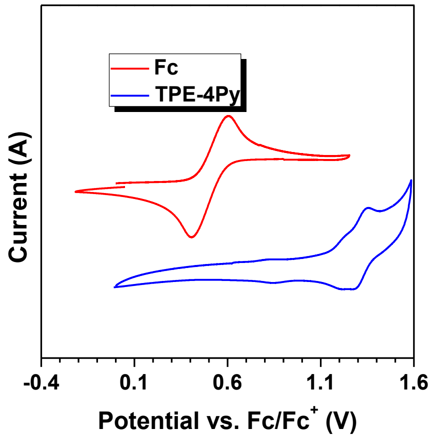

Figure S3. Cyclic voltammograms of TPE-4Py.

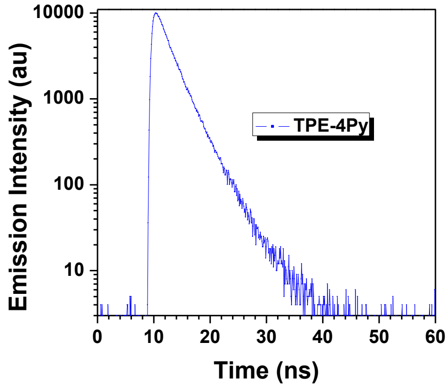

Figure S4. Photoluminescence (PL) decay curve of TPE-4Py powder.

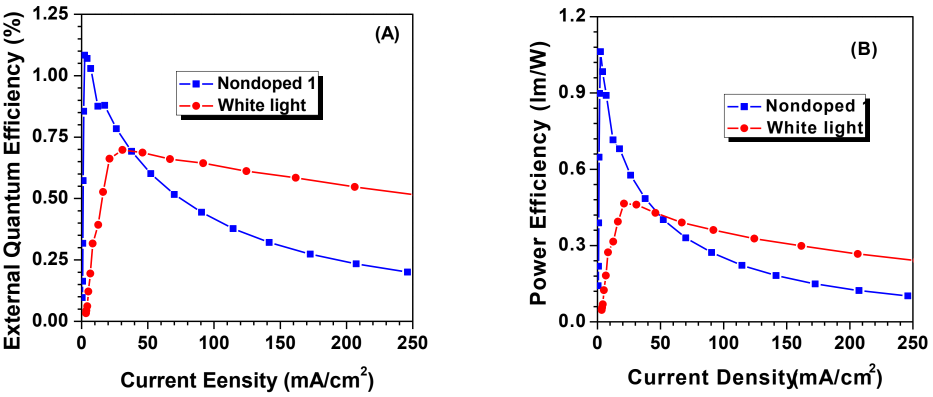

Figure S5 Changes in (A) external quantum efficiency with the current density, (B) power efficiency with the current density. Device configuration: Nondoped 1: ITO/PEDOT:PSS (30 nm)/Poly-TPD (30 nm)/TPE-4Py (30 nm)/TPBi (50 nm)/Liq (1 nm)/Al (100 nm); White light: ITO/PEDOT:PSS (30 nm)/Poly-TPD (30 nm)/mCP:OXD-7:TPE-4Py (70:20:10, 30 nm)/TPBi (50 nm)/Liq (1 nm)/Al (100 nm).

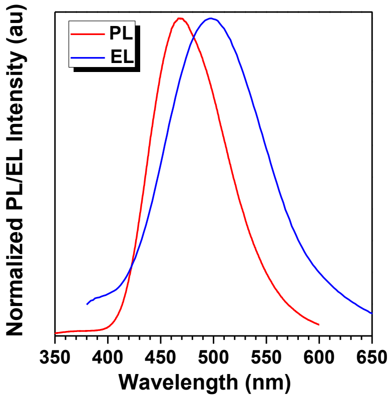

Figure S6 The PL spectrum of TPE-4Py film and EL spectrum of nondoped 1 OLED device, device configuration: Nondoped 1: ITO/PEDOT:PSS (30 nm)/Poly-TPD (30 nm)/TPE-4Py (30 nm)/TPBi (50 nm)/Liq (1 nm)/Al (100 nm)

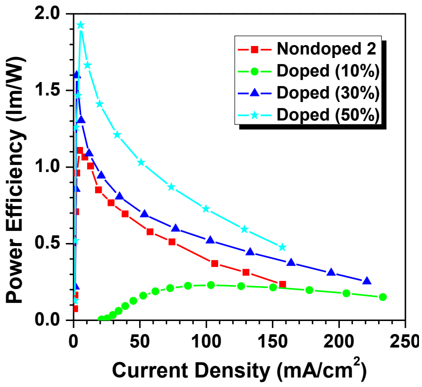

Figure S7 Changes in power efficiency with the current density. Device configuration: Nondoped 2: ITO/PEDOT:PSS (30 nm)/TPE-4Py (30 nm)/TmPyPB (50 nm)/Liq (1 nm)/Al (100 nm); Doped: ITO/PEDOT:PSS (30 nm)/mCP:TPE-4Py (10% or 30% or 50%, 30 nm)/TmPyPB (50 nm)/Liq (1 nm)/Al (100 nm).
